# Investigating primary school educators’ insights into attention deficit hyperactivity disorder: A cross sectional study in the Greater Accra Region, Ghana

**DOI:** 10.1371/journal.pmen.0000376

**Published:** 2025-09-03

**Authors:** Stephanie Eyram Diaba, Yaw Akye Essuman, Gurinder Singh, Delali Fiagbe

**Affiliations:** 1 University of Ghana Medical School, University of Ghana, Accra, Ghana; 2 Colchester East Hants Health Centre, Nova Scotia, Canada; 3 Department of Psychiatry, University of Ghana Medical School, University of Ghana, Accra, Ghana; PLOS: Public Library of Science, UNITED KINGDOM OF GREAT BRITAIN AND NORTHERN IRELAND

## Abstract

Schoolteachers play an integral role in the diagnosis and management of students with Attention Deficit/ Hyperactivity Disorder (ADHD). Despite its increased importance in Ghana, no study has been conducted to assess the knowledge and misperceptions of Ghanaian schoolteachers about ADHD. This study thus aimed to evaluate schoolteachers’ level of knowledge and misperceptions about ADHD. This was a descriptive cross-sectional study conducted among teachers from eleven primary schools in the Greater Accra Region of Ghana from February to July 2023. A validated and self-administered questionnaire, comprising a demographic questionnaire and the Knowledge of Attention Deficit Disorder Scale (KADDS), was used to collect data on participants’ sociodemographic characteristics, knowledge, and misperceptions about ADHD. Descriptive and inferential statistics were performed on the data using SPSS version. 26. Statistical significance was set at p = 0.05. The study included 170 participants, the majority being female (62.4%). The average percentage of knowledge regarding ADHD general knowledge, symptoms/diagnosis, and treatment was 32.29%, 40.92%, and 31.32%, respectively. The overall proportion of correct, incorrect, and “do not know” responses was 34.90%, 23.40%, and 41.70%, respectively. Common misconceptions included the belief that children with ADHD often outgrow the condition and that reducing dietary sugar alleviates symptoms. Educational background (p = 0.037), prior ADHD training (p = 0.005), and experience teaching an ADHD child (p = 0.003) were significantly associated with higher knowledge scores. There is a need for childhood mental health training among teachers and further research in this field in Ghana.

## Introduction

Attention Deficit-Hyperactivity Disorder is a common childhood neurodevelopmental disease that frequently persists into adulthood [1]. Described as a chronic disorder, it is often associated with other disorders such as anxiety and mood disorders, and eventually substance abuse [2].

The global prevalence rate of ADHD was found to be 5.29% [3], with an estimated 4%-12% of school children globally affected [4]. A study in the USA revealed a national prevalence of 12.9% among school children [5].

In comparison, the prevalence of ADHD in Ghana shows notable variability across different studies. A study conducted in a Ghanaian population of primary school children found ADHD to be the second major cause of child and adolescent mental health disorders, with a prevalence of 1.64% [6]. Other studies in other Ghanaian populations found prevalence rates of 5% [7], 7% [8], and 12.9% [9]. Studies indicate that ADHD may be rising by 6–10% in developing nations such as Ghana [10,11].

Currently, the causes and risk factors for ADHD are unknown - only genetic associations have been found [1]. Scientists are now investigating the role of brain injury, lead exposure, alcohol and tobacco use during pregnancy, premature delivery, and low birth weight [1,12].

Children who meet the diagnostic criteria for ADHD demonstrate behavioral symptoms that exceed the typical fluctuation expected for their age and level of intellectual functioning [13]. Although some studies have suggested that 50% of ADHD children outgrow their symptoms by adulthood [14,15], another study [16] in 2022 refuted these claims, indicating that 90% of ADHD adults continue to experience residual symptoms, albeit with intermittent periods of remission.

**Fig 1:**
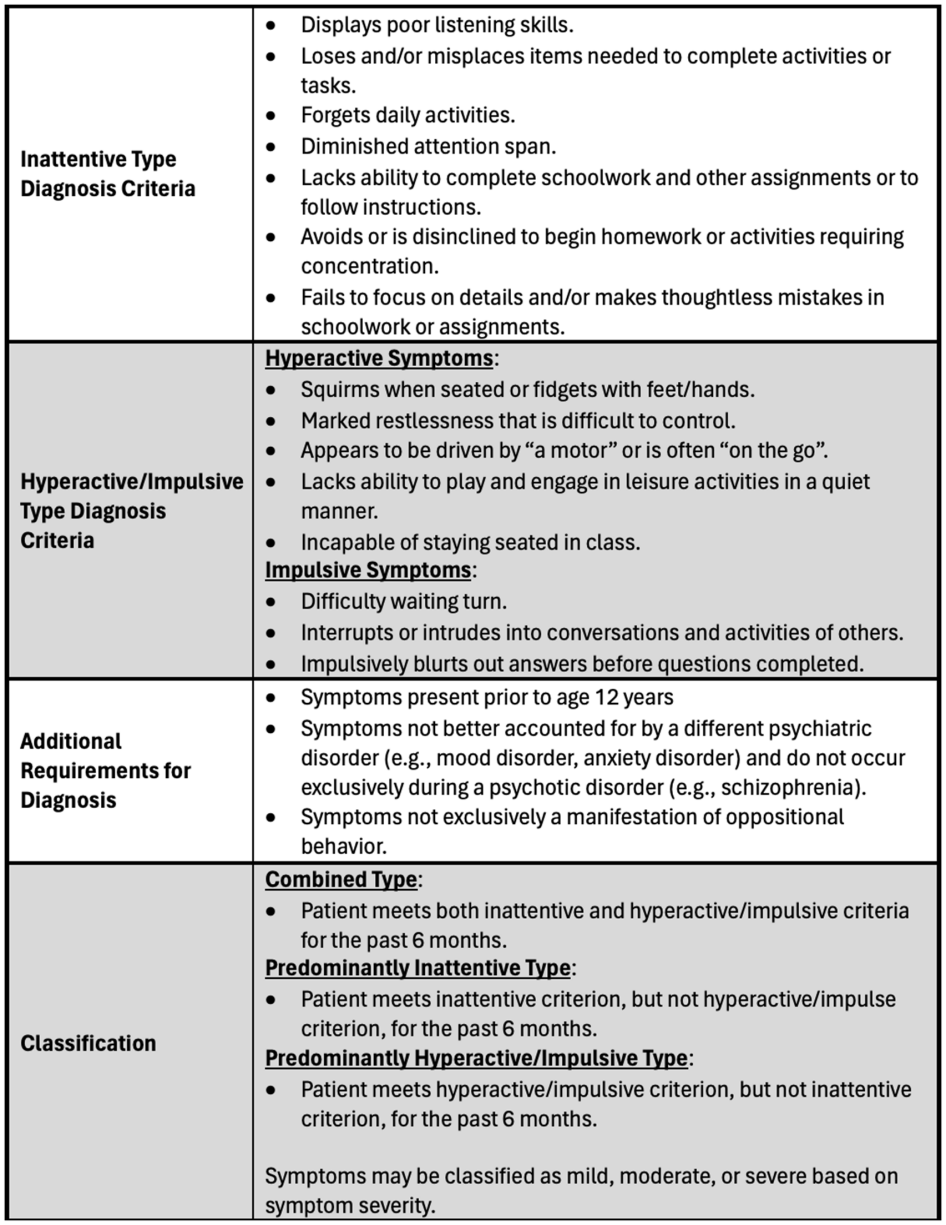
Classification and symptoms of ADHD according to the DSM-5 criteria.

Fig 1 shows the diagnostic criteria and symptoms of ADHD as defined by the DSM-5 Criteria. Attention Deficit-Hyperactivity Disorder is an exclusionary diagnosis given when a child under the age of 12 has symptoms that have persisted for at least six months and cause issues in two or more places (home, school, church) [2]. The symptoms should have had a negative influence on academic, social, and/or occupational performance [17]. Among other social and mental health difficulties, children with ADHD are more likely than their peers to fail to graduate from high school, finish college, have few or no friends, perform poorly at work, and engage in antisocial behavior [14].

Some treatment options for ADHD that are available include: behavior therapy, medication, and classroom management techniques. For those between 6 and 12 years, both behavioral therapy and medication are recommended [18]. Training in behavior management is recommended as the first line for parents of affected children less than 6 years [19].

A teacher is regarded as one of a child's key sources for developing social skills and learning fundamental life ideas [20]. Because of the amount of time they spend studying children, they may be critical in identifying ADHD [18]. Teachers also play an important role in ADHD management by using classroom management tactics such as favorable seating arrangements [21] and the use of suitable gestures [22], as well as referring cases and ensuring treatment compliance. Medical practitioners usually need teachers to provide ADHD behavioral assessments for pupils throughout the diagnostic process [2]. The success of ADHD classroom interventions is primarily based on teachers’ knowledge and perception of the disease, and as such, a lack of comprehension by teachers greatly hinders classroom behavior management programs [23].

Teachers often employ labels such as “ADHD” not as formal diagnoses but as pragmatic tools to interpret and manage classroom behaviors. This practice aligns with McMahon’s (2012) observation that teachers may use labeling to navigate classroom dynamics, distinguishing between medical diagnoses and practical classroom management strategies [24]. Rather than seeking clinical precision, teachers focus on observable behaviors that disrupt learning environments, using labels to communicate concerns and implement interventions. Blotnicky-Gallant et al. (2015) further emphasize that enhancing teachers’ practical strategies for managing ADHD-related behaviors can be more beneficial than solely increasing clinical knowledge [25].

Moreover, teachers’ understanding of ADHD can be influenced by local beliefs and educational pressures. Perold et al. (2010) observed that South African teachers often held misconceptions about ADHD, affecting their ability to support affected students effectively [26]. Therefore, understanding teachers’ knowledge and perceptions of ADHD involves recognizing their interpretations shaped by local values, educational pressures, and available support systems. This study acknowledges these complexities and situates its exploration of teachers’ knowledge within both biomedical and sociocultural frames.

While ADHD is widely recognized in biomedical literature as a neurodevelopmental disorder with global prevalence, there remains significant debate about its conceptualization and diagnosis across cultural contexts. Scholars such as Singh (2018) argue that ADHD reflects not only biological realities but also sociocultural interpretations of behavior, attention, and childhood norms [27]. Diagnostic practices rooted in frameworks like the DSM-5 may not fully capture behavioral expectations or coping strategies prevalent in low- and middle-income countries like Ghana. For instance, Afeti and Nyarko (2017) found a 12.8% prevalence of ADHD among primary school pupils in Ghana’s Hohoe Municipality, and highlighted the disorder’s impact on academic performance and the need for culturally sensitive diagnostic approaches [9].

Existing studies from diverse contexts reveal widespread gaps and misconceptions in teachers’ understanding of ADHD. For instance, Alkahtani (2013) and Al-Moghamsi & Aljohani (2018) reported that Saudi Arabian teachers held significant misunderstandings about ADHD’s causes and treatments [28,29]. Similarly, Perold et al. (2010) found that South African primary school teachers had limited knowledge, particularly about medication and long-term prognosis [26]. A study by Youssef et al. (2015) in Trinidad and Tobago revealed that while teachers’ factual knowledge was low, their willingness to learn was high, indicating an opportunity for effective training [30]. These findings underscore the global relevance of assessing teacher knowledge, particularly in under-researched contexts like Ghana, where limited resources make educators the first—and sometimes only—line of response to behavioral difficulties.

Few studies that have assessed the knowledge of ADHD among primary school teachers found a general lack of knowledge and significant misperceptions about the disease [28,29,31,32]. Despite growing interest in neurodevelopmental disorders, limited research has explored how primary school teachers in Ghana understand and respond to ADHD. Most existing studies on teacher knowledge originate from high-income or middle-income countries, with minimal representation from sub-Saharan Africa. Even within Ghana, available studies are not focused specifically on primary educators, who are often the first to observe behavioral challenges in the classroom. Furthermore, little is known about how misconceptions coexist with accurate knowledge, or how teachers’ lived experiences—such as previous contact with children with ADHD—influence their understanding. This study addresses these critical gaps by assessing the levels of knowledge, uncertainty, and misperception among teachers using a validated tool. It contributes to both local and global discussions on how best to support educators in identifying and managing ADHD in low-resource contexts.

In this study, the term “insights” encompasses teachers’ knowledge, beliefs, and misperceptions about ADHD, particularly regarding its causes, symptoms, and management. Therefore, throughout this study, “insight” includes both accurate knowledge and commonly held misconceptions about ADHD, and “Educators” refers specifically to classroom teachers working in primary schools.

This study aimed to investigate the following questions:

1. What is the primary school teachers’ level of knowledge of ADHD?
2. Is there a relationship between teachers’ level of knowledge of ADHD and certain sociodemographic characteristics?

## Materials and methods

### Ethics statement

The authors assert that all procedures contributing to this work comply with the ethical standards of the relevant national and institutional committees on human experimentation and with the Helsinki Declaration of 1975, as revised in 2013. All procedures involving human subjects/patients were approved by the University of Ghana Medical School Community Health Dissertation Review Committee [UGMS-CHDRC/077/2023]. Written informed consent was obtained from all subjects.

### Study area and design

This was a descriptive cross-sectional study conducted from February to July 2023, designed to assess the level of knowledge and misconceptions about ADHD among primary school teachers in selected schools in the Greater Accra Region of Ghana.

Participant recruitment period began on the 26th of April, 2023, and ended on the 4th of May 2023. It was carried out in eleven primary schools located in Accra and Tema, Ghana, namely: DEKS Preparatory School, Marbs International School, Twedaase Primary School, Padmore Street No.2 Primary School, Oninku Drive No.1 Primary School, First Baptist Church School, Aggrey Road No.1 Primary School, Star Basic School - all in Tema - and New Hope Primary School, Ministry of Health Basic School and Bishop Bowers School in Accra. The selected schools included a mix of public and private institutions located in both urban and peri-urban areas of Accra and Tema. Most schools had an average enrollment of 200–500 pupils and employed between 8–25 teaching staff. The schools were co-educational and followed the national Ghana Education Service curriculum.

### Sampling strategy and recruitment

Sample size was calculated using Cochran’s Formula [33], n =Z^2^P(1–P)/d^2^

Where n = sample size, p = prevalence of study variable, and d = margin of error.

For this study, the Confidence interval (CI) is 95%, Z score: 1.96, d = 5%, prevalence: 7% [8], Calculated Sample Size: 100, sample size used: 170.

A convenience sampling strategy was used to select the participating schools, all of which were regular schools. These schools were chosen because they had a history of supporting academic research, did not require prior clearance from the Ghana Education Service for data collection, and were located in proximity to the principal researcher and the research assistant. This approach facilitated timely access and ensured a smoother approval process at the school level, while still allowing for diversity across public and private institutions to capture a diverse cross-section of educational settings and teaching experiences.

### Study participants

Schools were included if they were officially registered, located within the Greater Accra Region, and provided education for children aged 6–12 years. Teachers were eligible to participate if they were currently employed full-time in one of the selected schools and had at least one year of teaching experience. Trainee teachers, part-time staff, and non-classroom staff were excluded.

Permission was sought from relevant authorities, such as school administrators, to conduct the study on their premises and with their teachers. Teachers in the individual schools were informed about the study and encouraged to pick up questionnaires if interested in participating—170 showed interest. A written informed consent form was signed by all participants thereafter.

### Data collection

A pretested, structured, self-administered questionnaire with close-ended questions was used to obtain information about participants. All questionnaires were distributed and collected in person. The primary researcher visited each school to explain the purpose of the study, distribute the forms, and ensure confidentiality. Interested teachers completed the questionnaires independently at a convenient time and returned them sealed in envelopes.

The questionnaire consisted of two parts. The first component assessed sociodemographic characteristics of participants, including age, gender, education level, education role, teaching experience with ADHD pupils, and years of teaching experience. The Knowledge of Attention Deficit Disorder Scale was used in the second section to ascertain the knowledge and perception of ADHD of participants. Sciutto and colleagues developed the understanding of the Knowledge of Attention Deficit Disorders Scale (KADDS) in 2000. It is one of the most extensively used tools to assess teachers’ understanding of ADHD, and has been adapted in other studies [34]. It is a 36-item questionnaire that assesses teachers’ knowledge in three areas: general ADHD knowledge (15 items), ADHD symptoms and diagnosis (9 items), and ADHD treatment (12 items). The items include statements about the causes and prevalence of ADHD, observable classroom symptoms like inattention or hyperactivity, and treatment methods such as stimulant medication and behavioral therapy. The tool also includes questions designed to identify common myths, such as beliefs about sugar intake or the use of electroconvulsive therapy.

Each KADDS item is stated in terms of an ADHD statement and has a true (T), false (F), or “do not know” (DK) response format. This approach helped teachers to distinguish between what they did not know and an inaccurate belief or perception. The KADDS scale reported a Cronbach’s alpha of 0.71 for each subscale and 0.86 for the general scale, so a suitable internal consistency is assumed [34].

### Data entry and analysis

The data entry was done using Microsoft Excel version 13, and any inconsistency was rectified. The data were analyzed using Statistical Package for Social Sciences (SPSS ver. 26, IBM Corp., Armonk, NY, USA). Descriptive statistics such as frequencies and percentages were used to describe the variables under study, and the data were presented in charts, graphs, and tables. Chi-square test was used to analyze the association between sociodemographic characteristics of participants, and knowledge and misperceptions about ADHD. P-values <0.05 were considered statistically significant.

A multivariate analysis was used to assess the knowledge of participants. Here, knowledge was assessed by correct answers provided, whereas misperceptions were assessed by wrong answers. “Do not know” responses, indicating a lack of knowledge, were also analyzed [26,30]. Participants were given a mark for each correct answer provided and no mark for incorrect and “do not know” responses. The maximum total score that could be achieved was 36. The overall mean knowledge score was graded into poor (0.0-12.0), moderate (12.1-24.0), and good (24.1-36.0), and the scores were subsequently converted into percentages.

## Results

This study examined the knowledge and misperceptions about ADHD of primary school teachers in the Greater Accra Region of Ghana.

### Sociodemographic Characteristics

Table 1 provides a summary of the sociodemographic characteristics of participants in this study (Table 1).

**Table 1:** Sociodemographic characteristics of study participants (N = 170).

Characteristics	Frequency (N)	Percentage (%)
**Sex**		
Male	64	37.6
Female	106	62.4
**Age**, years		
25-29	19	11.2
30-34	23	13.5
35-39	14	23.5
40-44	31	18.2
45-49	36	21.2
50+	21	12.4
**Duration of teaching**, years		
0-10	57	33.5
11-20	68	40
21-30	39	22.9
>30	6	3.5
**Education**		
University	58	34.1
Training institute	111	65.3
Not formally trained	1	0.6
**The belief that children can have mental health disorders**		
Yes	165	97.1
No	5	2.9
**ADHD training**		
Yes	97	57.1
No	73	42.9
**Taught a child with ADHD**		
Yes	115	67.6
No	55	32.4

ADHD = Attention Deficit Hyperactivity Disorder.

The majority (165, 97.1%) believed that children could have mental health disorders. Most teachers (165, 97.1%) answered that they had taught a child with a mental health disorder, with 64 of them (50%) suspecting behavioral disorders and 56 (43.7%) suspecting developmental disorders (Table 1). With regards to ADHD training, 97 teachers (57.1%) answered that they had received it, whilst 73 (42.9%) said they had not received any training (Table 1). One hundred and fifteen teachers (67.6%) had taught a child with ADHD before, but 55 (32.4%) had not (Table 1).

Table 2 outlines the relationship between the knowledge of ADHD and various sociodemographic characteristics of study participants (Table 2). Significant associations were found between the level of knowledge and educational background (*P* = 0.037), prior ADHD training (P =0.005), and experience teaching an ADHD child (*P* = 0.003) (Table 2).

**Table 2:** Sociodemographic characteristics against the level of knowledge of participants (N = 170).

Characteristics	Level of knowledge			X^2^	df	P-value
	Poor (%)	Moderate (%)	Good (%)			
**Sex**				1.87	2	0.393
Male	33 (19.4)	30 (17.6)	1 (0.6)			
Female	44 (25.9)	61 (35.9)	1 (0.6)			
Total	77 (45.3)	91 (53.5)	2 (1.2)			
**Age**, years				6.039	10	0.812
25-29	8 (4.7)	11 (6.5)	0 (0.0)			
30-34	11 (6.5)	11 (6.5)	1 (0.6)			
35-39	19 (11.2)	20 (11.8)	1 (0.6)			
40-44	16 (9.4)	15 (8.7)	0 (0.0)			
45-49	16 (9.4)	20 (11.8)	0 (0.0)			
50+	7 (4.1)	14 (8.2)	0 (0.0)			
Total	77 (45.3)	91 (53.5)	2 (1.2)			
**Duration of teaching**, years				3.934	6	0.686
0-10	24 (14.1)	33 (19.4)	0 (0.0)			
11-20	33 (19.4)	33 (19.4)	2 (1.2)			
21-30	17 (10.0)	22 (12.9)	0 (0.0)			
>30	3 (1.8)	3 (1.8)	0 (0.0)			
Total	77 (45.3)	91 (53.5)	2 (1.2)			
**Educational background**				10.197	4	0.037*
University	35 (20.6)	23 (13.5)	0 (1.2)			
Training institute	41 (24.1)	68 (40.0)	2 (1.2)			
Not formally trained	1 (0.6)	0 (0.0)	0 (0.0)			
Total	77 (45.3)	91 (53.5)	2 (1.2)			
**The belief that children can have mental health disorders**				0.133	2	0.936
Yes	75 (44.1)	88 (51.7)	2 (1.2)			
No	2 (1.2)	3 (1.8)	0 (0.0)			
Total	77 (45.3)	91 (53.5)	2 (1.2)			
**ADHD training**				10.432	2	0.005*
Yes	34 (20.0)	61 (35.9)	2 (1.2)			
No	43 (25.3)	30 (17.6)	0 (0.0)			
Total	77 (45.3)	91 (53.5)	2 (1.2)			
**Taught a child with ADHD**				11.471	2	0.003*
Yes	42 (24.7)	71 (41.7)	2 (1.2)			
No	35 (20.6)	20 (11.8)	0 (0.0)			
Total	77 (45.3)	91 (53.5)	2 (1.2)			

ADHD = Attention Deficit Hyperactivity Disorder; Statistically significant associations

### Knowledge level of ADHD using the KADDS tool

The overall mean knowledge score was 12.35 ± 6.57 (34.31%). Most participants (53.5%) had moderate knowledge about ADHD, while 45.3% had poor knowledge, and only 1.2% had good knowledge (Table 2). Table 3 summarizes the correct, incorrect, and “do not know” responses across the three KADDS subscales (Table 3).

**Table 3:** Responses across the three KADDS subscales (N = 170).

Subscale	Correct Responses (%)	Incorrect Responses (%)	Do Not Know Responses (%)
General Knowledge	32.39	27.69	39.92
Symptoms and Diagnosis	40.92	21.76	37.32
Treatment	31.32	20.78	47.90
**Total**	34.90	23.40	41.70

The general knowledge subscale evaluated their knowledge about epidemiology, common myths, and non-specific information about ADHD. The second subscale includes 9 items assessing the symptoms and diagnosis of ADHD, and the third contains 12 items about the treatment of ADHD.

**Fig 2:**
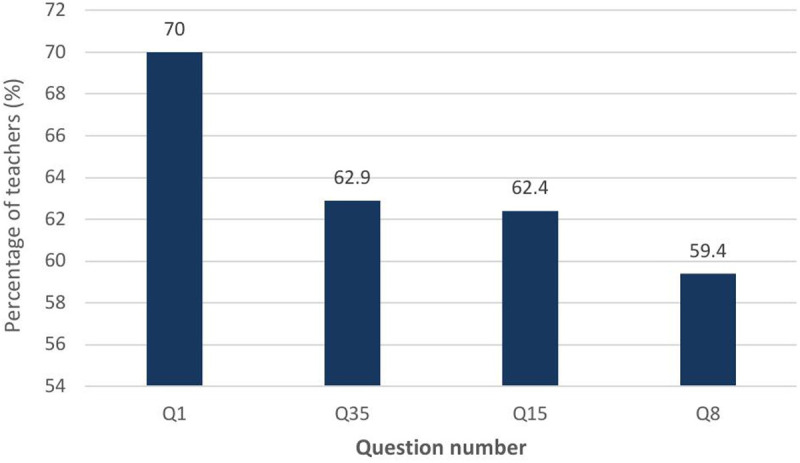
Top Four Items with Highest “Do Not Know” Responses.

Item 1 was from the general knowledge subscale and suggested that ADHD occurs in approximately 15% of school-age children. One hundred and nineteen teachers (70%) did not know this was false (Fig. 2).

Item 8 was from the treatment subscale, and 101 (59.4%) did not know that the statement “Antidepressant drugs have been effective in reducing symptoms for many ADHD children” was true (Fig. 2). Item 35, also from the treatment subscale, revealed 107 (62.9%) did not know the statement “Electroconvulsive Therapy (i.e., shock treatment) has been found to be an effective treatment for severe cases of ADHD” was false (Fig. 2). Item 15, which states the side effects of stimulant drugs used for treatment of ADHD may include mild insomnia and appetite reduction was true, but 106 teachers (62.4%) did not know this (Fig 2). Notably, 52.4% of participants incorrectly believed that ADHD students are more likely to have trouble in new situations than non-ADHD students. A significant proportion (42.9%) incorrectly believed that reducing dietary sugar intake is effective in reducing ADHD symptoms.

## Discussion

This study aimed to evaluate the knowledge and misperceptions about ADHD in primary school teachers in Ghana and assess the predictors of knowledge. In this study, we not only found that ADHD knowledge was moderate but that there were several misperceptions about the general knowledge, symptoms, diagnosis, and treatment of ADHD.

The participants in this study had a moderate level of knowledge (34.9%). This may be attributable to a disbelief in the legitimacy of the disorder and a belief that it is a means for parents to rationalize their children’s shortcomings [35,36]. Our findings are similar to a study in Madinah, Saudi Arabia, which reported an overall knowledge score of 38% among its participants [29]. The outcomes of the current study are somewhat inferior to those seen in Saudi Arabia (17.2%), the USA (47.8%), and Cape Town (42.6%) [26,28,34]. Another study in Australia [37] revealed that 25% of participants had high knowledge, which is more than in this study (1.2%). This is likely because all participants in the Australian study had prior experience in teaching at least one ADHD student, whereas in the current study, only 24.7% had experience.

### Predictors of the Level of ADHD Knowledge

The following variables were found to be predictors of the level of knowledge of ADHD in the teachers: educational background *(P* = 0.037), prior training in ADHD (*P = 0.005*), and previous experience in teaching an ADHD child (*P* = 0.003). This is similar to studies that also found a significant association between the level of knowledge of ADHD and prior teaching and experience with ADHD of participants [28,29].

Our findings must be interpreted in the context of how ADHD is constructed and managed in Ghanaian schools. Rather than viewing teachers’ responses strictly in terms of clinical correctness, it is important to consider their roles as pragmatic actors navigating behavioral challenges in under-resourced settings.

Teachers may invoke ADHD-related labels or behaviors not to diagnose but to manage classroom conduct, refer students, or explain academic difficulties to parents. As Singh (2018) and Bröer & Agyekum (2020) suggest, such practices reflect not misinformation, but adaptive strategies in environments where formal diagnostic support may be limited or absent [27,38].

Furthermore, in the Ghanaian setting where school counselors and child psychiatrists are sparse, teachers’ basic awareness and recognition of behavioral challenges—even without detailed clinical accuracy—can play a vital role in initiating support for children.

Access to formal support services such as school counselors, child psychologists, and pediatric mental health referral systems is extremely limited. Most public schools do not have in-house counseling services, and referrals often rely on parental initiative or external advocacy. A study evaluating guidance and counseling units in senior high schools in the Okere District of Ghana reported that the implementation of such services is often hindered by these constraints [39]. In such settings, teachers serve as critical intermediaries between students and any potential help. Their ability to recognize behavioral patterns associated with ADHD—whether or not they label them accurately—can be the difference between a child being supported or left to struggle in silence. For instance, teachers who are aware of ADHD-related behaviors may be more likely to advise parents to seek further evaluation at hospitals or clinics. Conversely, misperceptions or lack of awareness may lead to punitive responses or underreporting. Therefore, building teachers’ practical knowledge of ADHD is not just educational—it is a matter of increasing access to care in systems where no formal mental health screening pathways exist.

This aligns with findings from Woyessa et al. (2019), who reported that Ethiopian primary school teachers often held misconceptions about ADHD, underscoring the need for targeted training programs [40]. Similarly, Youssef et al. (2015) highlighted that Trinidadian teachers’ knowledge about ADHD was low, but their positive attitudes toward the disorder suggest openness to further education and training [30].

It is important to consider the implications of relying on Western frameworks such as the DSM-5 to understand ADHD in the Ghanaian context. While the DSM-5 provides structured diagnostic criteria, these are shaped by sociocultural norms that may not fully align with those in Ghana. Behavioral expectations in Ghanaian schools, the level of tolerance for hyperactivity or inattentiveness, and the interpretation of discipline and academic performance can differ significantly from those in high-income settings where these criteria were developed. Therefore, applying these standards without adaptation may risk overlooking context-specific expressions of distress or mislabelling culturally normative behavior as pathological. This reinforces the need for future studies to investigate how ADHD manifests locally and how teachers interpret and respond to it in ways that are meaningful and relevant within their own educational environments.

### General Knowledge subscale

The general knowledge subscale had a high proportion of “do not know” responses (39.92%), indicating a lack of general knowledge about ADHD. About half of the participants (50%) did not know whether children outgrow their ADHD symptoms (item 19), whilst only 27.6% correctly identified that they, in fact, do not outgrow their symptoms. In a study in Saudi Arabia, 53.1% of teachers knew that students do not outgrow their symptoms [29]. Holding this view could lead teachers to overlook the seriousness of the disorder. This disparity is not expected since more participants in our study (67.6%) reported prior experience in teaching an ADHD child. This reveals a possible gap in the training received by teachers. A large majority of the participants also did not know that the prevalence of ADHD was less than 15% (item 1). This may be explained by the fact that it may be too much of an ask for a teacher to know the specific prevalence rate of ADHD, as unpopular a disease in national circles as it is.

This subscale also recorded the highest proportion of misperceptions (27.69%), i.e., incorrect answers, among all subscales. For instance, most participants (52.4%) incorrectly believed that ADHD students were more likely to have trouble in new situations than non-ADHD students (item 27). This is a slightly lower percentage than the 69.7% revealed by a study in Saudi Arabia [29]. Even though 57.1% of them admitted to having received training in the identification of ADHD, the low percentage of correct answers to item 27 suggests that additional training is required to improve the quality of knowledge about ADHD. Also, most participants (42.9%) incorrectly believed that a child who is more attentive in front of video games will also be able to remain attentive for at least an hour of class or homework (item 22). This indicates that the majority of teachers have misconceptions about the change of ADHD symptoms across different tasks and settings. This is problematic because ADHD children perform better in tasks they have chosen themselves and struggle to remain attentive in front of repetitive work, and as such, it would be important for teachers to present school work and homework in a way that is engaging and interesting to the student. Participants, however, did have a good proportion of correct responses to some questions under the general knowledge subscale. Many of them (57.7%) were able to correctly identify that depression is more likely in children with ADHD than those without (item 17). An encouraging finding, this advocates for further training of teachers to be adequately equipped to identify associated mental health disorders, such as anxiety, and subsequently to support the affected child. Also, a high level of knowledge was found in response to item 32. Here, 64.1% of the participants correctly identified that the majority of children with ADHD had some level of academic difficulty, whereas 18.8% had the misperception that they did not. Students with ADHD have substantially lower achievement in reading, writing, and numeracy and thus need great support to manage their inattention, impulsivity, and hyperactivity [41]. Knowledge about this will help teachers to appropriately use resources at their disposal to give ADHD children a better academic experience and, eventually, a better quality of life as adults.

### Symptoms and diagnosis subscale

Participants had the highest proportion of correct responses (40.92%) on the symptoms and diagnosis subscale of the KADDS questionnaire. This is similar to results reported by other studies [28,42]. Half of the participants (50.0%) correctly identified that children with ADHD are distracted easily (item 3), and 57.7% recognized that ADHD children fidget and squirm in their seats (item 9). This suggests that a considerable number of teachers can identify students who are likely affected by ADHD. Although these symptoms are of little predictive value, a diagnosis cannot be made in their absence [43]. When asked about the need for ADHD symptoms to be present in two or more settings for a diagnosis (item 21), 67% of participants correctly identified this as the truth. This is crucial because teachers’ observations allow them to educate parents about ADHD and encourage parents to look out for similar behaviors in other settings.

However, 52.4% did not know that a diagnosis of ADHD can only be made when symptoms have been present before age 12 (item 5). This is concerning because primary school teachers interact with this age group of children. Children with ADHD should be diagnosed as soon as feasible and placed in a customized educational program that builds on their strengths over time to lessen the negative effects of subpar academic achievement [44].

Unfortunately, 45.9% and 40% of participants incorrectly believed that ADHD children have an inflated sense of self-esteem (item 11) and a history of being destructive towards others’ possessions (item 14), respectively. These are dangerous misconceptions to have because they harm students’ self-esteem, future achievements, and social relationships [45].

### Treatment of ADHD

The subscale on ADHD treatment recorded the highest proportion of “do not know” responses (47.90%) in this study. Information about the medical treatment of ADHD may be uncommon in their circles, and this may explain why, when asked about the use and side effects of antidepressants (item 8), stimulant medications (item 15), and electroconvulsive therapy, the majority of participants, i.e., 59.4%, 62.4% and 62.9%, respectively, did not know.

The highest proportion of incorrect responses (39.4%) was on item 23, which questioned whether reducing dietary sugar intake was effective in reducing ADHD symptoms. This indicates that almost two-fifths of participants had misperceptions about the role of diet in ADHD symptoms. Although a study conducted in the middle region of Saudi Arabia, similarly found that this item had the highest proportion of incorrect responses (26.8%) among its respondents [28], another study, also in another Saudi population in Madinah City, contrastingly found that 77.2% of their respondents had correctly denied the effectiveness of reducing dietary sugar in reducing ADHD symptoms [29]. This mistaken belief could lead to wrong suggestions to reduce the sugar intake of children in a bid to reduce such symptoms, leading to impaired growth from poor nutrition [34]. It is important to provide correct information to teachers regarding the treatment of ADHD to enable them to disregard harmful misinformation peddled in the absence of the right information. In the past few years, myths and misconceptions about COVID vaccines containing 5G technology [46] and vaccines causing autism [47] had to be dispelled with the right information to prevent missing out on the benefits of those vaccines.

The highest proportion of correct responses (68.8%) was on item 10, which highlighted the effectiveness of combining parent and teacher training in ADHD management with the use of medications. This is similar to findings in another study, which also found the highest proportion of correct responses on this item, albeit much lower (26.3%) than in this study [28]. This is an important finding as it means that most teachers acknowledge the significance of their involvement in the management of ADHD and may, thus, be willing to engage in training sessions on the topic.

### Limitations

One limitation of this study is its reliance on the DSM-5 framework and the KADDS tool, both developed within a Western, specifically U.S., psychiatric tradition. While these tools provide structured criteria for assessing ADHD knowledge, they reflect culturally specific assumptions about behavior, childhood, and disorder. In adopting them, our study participates in the broader global trend of applying standardized psychiatric classifications in non-Western contexts. As emphasized in transcultural psychiatry, such tools may not fully align with local norms or experiences of behavior and distress [48]. Therefore, our findings should be interpreted with caution, recognizing that the knowledge assessed is shaped by global definitions that may not fully capture culturally grounded understandings of ADHD in Ghana.

Other limitations of this study include the use of purposive sampling, which increases the possibility of researcher bias and a narrower generalization. Also, while the majority reported receiving some training in ADHD, the quality of the training could not be assessed and can reasonably be questioned, given that the level of knowledge was low.

## Conclusions

Schoolteachers are essential figures in the support, diagnosis, and management of ADHD children. This study sought to determine these teachers’ level of knowledge and misperceptions about ADHD. We found that the level of knowledge was moderate, indicating a lack of knowledge about ADHD. Teachers’ educational background, prior ADHD training, and experience in teaching an ADHD child were shown to be predictors of the level of ADHD knowledge.

Opportunities to provide sound theoretical knowledge and practical skills for all schoolteachers should be explored. These may include: creating a comprehensive mental health Continuing Professional Development (CPD) module, integrating comprehensive ADHD training into teacher education programs at colleges and universities, organizing mandatory in-service training for current teachers on ADHD using workshops and online courses, developing and distributing educational materials, such as guides and toolkits tailored to the Ghanaian context. Professional development should prioritize equipping teachers with effective classroom management techniques and fostering an understanding of ADHD that is grounded in everyday teaching experiences. This approach acknowledges the teachers’ role as frontline responders to behavioral challenges, emphasizing the value of their contextual insights in supporting students with attention and hyperactivity difficulties. This will improve their level of knowledge of ADHD and mental health disorders in general. This study highlights the limited knowledge and persistent misconceptions about ADHD among primary school teachers in Ghana. As key frontline observers of student behavior, these educators play a critical role in the early identification and support of children with neurodevelopmental challenges. Yet, their understanding is often shaped by a mix of accurate information, uncertainty, and cultural and religious beliefs. By addressing a significant research gap in a sub-Saharan African context, this study contributes to a more global understanding of ADHD literacy and the educational systems that support—or fail—students with behavioral needs.

Using DSM-based tools to assess Ghanaian teachers’ knowledge of ADHD introduces a specific biomedical framework that may not align with local conceptions of childhood behavior. Rather than evaluating their knowledge as correct or incorrect, it’s more productive to understand how global mental health concepts—such as ADHD—are interpreted, adapted, or resisted within specific cultural contexts. Transcultural psychiatry encourages us to engage with these differences and create mental health interventions that are both scientifically grounded and culturally meaningful. This reinforces the idea that in contexts like Ghana, what matters most is not diagnostic precision but the teacher’s ability to recognize behaviors, respond supportively, and connect families to whatever informal or formal resources may be available—even if those are limited.

Future interventions should prioritize practical, culturally sensitive training programs and policies that empower teachers as informed, confident advocates for their students.

## Supporting information

S1 Data This file contains the anonymized raw data collected from primary school teachers for the study titled “Knowledge and Perception of ADHD Amongst Primary School Teachers.” The dataset includes participants’ demographic information (e.g., age, gender, years of teaching experience, and prior training in ADHD), followed by their responses to the Knowledge of Attention Deficit Disorders Scale (KADDS). Each item response from the KADDS is recorded, along with total scores for knowledge, misconceptions, and general perceptions related to ADHD.
(SAV)

## References

[1] Faraone SV, Banaschewski T, Coghill D, Zheng Y, Biederman J, Bellgrove MA, et al. The World Federation of ADHD International Consensus Statement: 208 Evidence-based conclusions about the disorder. Neurosci Biobehav Rev. 2021;128:789–818. doi: 10.1016/j.neubiorev.2021.01.022 33549739 PMC8328933

[2] American Psychiatric Association. Diagnostic and statistical manual of mental disorders fifth edition text revision. 5th ed. American Psychiatric Association Publishing. 2022.

[3] Polanczyk G, de Lima MS, Horta BL, Biederman J, Rohde LA. The Worldwide Prevalence of ADHD: a systematic review and metaregression analysis. Am J Psychiat. 2007;164(6):942–8. doi: 10.1176/ajp.2007.164.6.942 17541055

[4] Wilens TE, Spencer TJ. Understanding attention-deficit/hyperactivity disorder from childhood to adulthood. Postgrad Med. 2010;122(5):97–109. doi: 10.3810/pgm.2010.09.2206 20861593 PMC3724232

[5] Zgodic A, McLain AC, Eberth JM, Federico A, Bradshaw J, Flory K. County-level prevalence estimates of ADHD in children in the United States. Ann Epidemiol. 2023;79:56–64. doi: 10.1016/j.annepidem.2023.01.006 36657694 PMC10099151

[6] Kusi-Mensah K, Donnir G, Wemakor S, Owusu-Antwi R, Omigbodun O. Prevalence and patterns of mental disorders among primary school age children in Ghana: correlates with academic achievement. J Child Adolesc Ment Health. 2019;31(3):214–23. 31805836 10.2989/17280583.2019.1678477

[7] Anokye R, Acheampong E, Edusei A, Owusu I, Mprah WK. Prevalence of attention-deficit/hyperactivity disorder among primary school children in Oforikrom, Ghana based on the disruptive behavior disorders rating scale. East Asian Arch Psychiatry. 2020;30(3):88–90. doi: 10.12809/eaap1907 32994377

[8] Ntiakoh-Ayipah D, Dogbe JA, Opoku MP, Twum F, Owusu M, Kumi H, et al. Prevalence of attention deficit hyperactivity disorder among pupils in primary schools in Ghana. J Int Special Needs Educ. 2018;23(2):69–78. doi: 10.9782/18-00011

[9] Afeti K, Nyarko SH. Prevalence and effect of attention-deficit/hyperactivity disorder on school performance among primary school pupils in the Hohoe Municipality, Ghana. Ann Gen Psychiatry. 2017;16:11. doi: 10.1186/s12991-017-0135-5 28228839 PMC5307701

[10] Kashala E, Tylleskar T, Elgen I, Kayembe KT, Sommerfelt K. Attention deficit and hyperactivity disorder among school children in Kinshasa, Democratic Republic of Congo. Afr Health Sci. 2005;5(3):172–81. doi: 10.5555/afhs.2005.5.3.172 16245986 PMC1831926

[11] Ofovwe CE, Ofovwe GE, Meyer A. The prevalence of attention-deficit/hyperactivity disorder among school-aged children in Benin City, Nigeria. J Child Adolesc Ment Health. 2006;18(1):1–5. doi: 10.2989/17280580609486611 25865095

[12] Thapar A, Cooper M, Jefferies R, Stergiakouli E. What causes attention deficit hyperactivity disorder? Arch Dis Child. 2012;97(3):260–5. doi: 10.1136/archdischild-2011-300482 21903599 PMC3927422

[13] WHO. International Classification of Diseases, Eleventh Revision (ICD-11). 2022. https://icd.who.int/browse11

[14] Barkley RA. International consensus statement on ADHD. January 2002. Clin Child Fam Psychol Rev. 2002;5(2):89–111. doi: 10.1023/a:1017494719205 12093014

[15] Sibley MH, Swanson JM, Arnold LE, Hechtman LT, Owens EB, Stehli A, et al. Defining ADHD symptom persistence in adulthood: optimizing sensitivity and specificity. J Child Psychol Psychiatry. 2017;58(6):655–62. doi: 10.1111/jcpp.12620 27642116 PMC5809153

[16] Sibley MH, Arnold LE, Swanson JM, Hechtman LT, Kennedy TM, Owens E, et al. Variable patterns of remission from ADHD in the multimodal treatment study of ADHD. AJP. 2022;179(2):142–51. doi: 10.1176/appi.ajp.2021.21010032 PMC8810708 34384227

[17] Sadock BJ, Sadock VA, Ruiz P, Kaplan HI. Kaplan & Sadock’s synopsis of psychiatry: behavioral sciences, clinical psychiatry; [Includes interactive eBook with complete content; updated with DSM-5!]. 11 ed. Philadelphia, PA: Wolters Kluwer. 2015.

[18] Alzahrani RH, Abd El-Fatah NK. Factors Affecting Knowledge of Attention-Deficit Hyperactivity Disorder Among Female Primary Schoolteachers in Taif City, Saudi Arabia. Cureus [Internet]. 2023; Available from: https://www.cureus.com/articles/161168-factors-affecting-knowledge-of-attention-deficit-hyperactivity-disorder-among-female-primary-schoolteachers-in-taif-city-saudi-arabia 10.7759/cureus.40057 PMC10325820 37425535

[19] Feng M, Xu J, Zhai M, Wu Q, Chu K, Xie L, et al. Behavior management training for parents of children with preschool ADHD based on parent-child interactions: a multicenter randomized controlled, follow-up study. Behav Neurol. 2023;2023:3735634. doi: 10.1155/2023/3735634 37727252 PMC10506873

[20] Samanci O. Teacher views on social skills development in primary school students. Educ. 2010;131(1):147.

[21] Gremmen MC, van den Berg YHM, Segers E, Cillessen AHN. Considerations for classroom seating arrangements and the role of teacher characteristics and beliefs. Soc Psychol Educ. 2016;19(4):749–74. doi: 10.1007/s11218-016-9353-y

[22] Geng G. Investigation of teachers’ verbal and non-verbal strategies for managing attention deficit hyperactivity disorder (ADHD) students’ behaviours within a classroom environment. Aus J Teacher Educ. 2011;36(7). doi: 10.14221/ajte.2011v36n7.5

[23] Pfiffner L, Barkley RA, DuPaul GJ. Treatment of ADHD in school settings. In: Barkley RA, editor. Attention deficit hyperactivity disorder: A handbook for diagnosis and treatment. 3rd ed. New York: Guilford Press. 2006. p. 547–89.

[24] McMahon SE. Doctors diagnose, teachers label: the unexpected in pre-service teachers’ talk about labelling children with ADHD. Int J Inclus Educ. 2012;16(3):249–64.

[25] Blotnicky-Gallant P, Martin C, McGonnell M, Corkum P. Nova Scotia teachers’ ADHD knowledge, beliefs, and classroom management practices. Can J Sch Psychol. 2015;30(1):3–21.

[26] Perold M, Louw C, Kleynhans S. Primary school teachers’ knowledge and misperceptions of attention deficit hyperactivity disorder (ADHD). S Afr J Educ. 2010;30:457–73.

[27] Singh I, Bergey MR, Filipe AM, Conrad P. ADHD in the United Kingdom: conduct, class, and stigma. Global Perspectives on ADHD. 2018.

[28] Alkahtani KDF. Teachers’ knowledge and misconceptions of attention deficit/hyperactivity disorder. Psychology. 2013;04(12):963–9. doi: 10.4236/psych.2013.412139

[29] Al-Moghamsi EY, Aljohani A. Elementary school teachers’ knowledge of attention deficit/hyperactivity disorder. J Family Med Prim Care. 2018;7(5):907–15. doi: 10.4103/jfmpc.jfmpc_183_18 30598932 PMC6259519

[30] Youssef MK, Hutchinson G, Youssef FF. Knowledge of and attitudes toward ADHD among teachers. Sage Open. 2015;5(1). doi: 10.1177/2158244014566761

[31] Bolinger SJ, Mucherah DrW, Markelz DrAM. Teacher knowledge of attention-deficit/hyperactivity disorder and classroom management. J Special Education Apprenticeship. 2020;9(1). doi: 10.58729/2167-3454.1098

[32] Weyandt LL, Fulton KM, Schepman SB, Verdi GR, Wilson KG. Assessment of teacher and school psychologist knowledge of Attention‐Deficit/Hyperactivity Disorder. Psychol Schools. 2009;46(10):951–61. doi: 10.1002/pits.20436

[33] Cochran WG. Sampling techniques. 3rd ed. New York: John Wiley & Sons. 1977.

[34] Sciutto MJ, Terjesen MD, Frank ASB. Teachers’ knowledge and misperceptions of Attention-Deficit/Hyperactivity Disorder. Psychol Sch. 2000;37(2):115–22.

[35] Bateman B. Learning disabilities: the changing landscape. J Learn Disabil. 1992;25(1):29–36. doi: 10.1177/002221949202500105 1740636

[36] Gargiulo RM, Bouck EC. Special education in contemporary society: an introduction to exceptionality. 6th ed. Los Angeles: SAGE Publications. 2018.

[37] Ohan JL, Cormier N, Hepp SL, Visser TAW, Strain MC. Does knowledge about attention-deficit/hyperactivity disorder impact teachers’ reported behaviors and perceptions? School Psychol Quart. 2008;23(3):436–49. doi: 10.1037/1045-3830.23.3.436

[38] Bröer C, Agyekum HA. Medicalization and manhood: Is an ADHD diagnosis emerging for allegedly troublesome boys in Accra, Ghana? Soc Sci Med. 2021;291:114465. doi: 10.1016/j.socscimed.2021.114465 34687961

[39] Mante DA, Maosen L. Evaluating the impact of counseling services in senior high schools in Ghana: The case of Okere district. Br J Educ. 2021;9.

[40] Woyessa AH, Tharmalingadevar TP, Upashe SP, Diriba DC. Primary school teachers’ misconceptions about Attention Deficit/Hyperactivity Disorder in Nekemte town, Oromia region, Western Ethiopia. BMC Res Notes. 2019;12(1):524. doi: 10.1186/s13104-019-4573-9 31429813 PMC6701197

[41] Lawrence D, Houghton S, Dawson V, Sawyer M, Carroll A. Trajectories of academic achievement for students with attention-deficit/hyperactivity disorder. Br J Educ Psychol. 2021;91(2):755–74. doi: 10.1111/bjep.12392 33259064

[42] Munshi A. Knowledge and misperceptions towards diagnosis and management of attention deficit hyperactive disorder (ADHD) among primary school and kindergarten female teachers in Al-Rusaifah district, Makkah City, Saudi Arabia. Int J Med Sci Public Health. 2014;3(4):444. doi: 10.5455/ijmsph.2014.120220141

[43] Pelham WE Jr, Gnagy EM, Greenslade KE, Milich R. Teacher ratings of DSM-III-R symptoms for the disruptive behavior disorders. J Am Acad Child Adolesc Psychiatry. 1992;31(2):210–8. doi: 10.1097/00004583-199203000-00006 1564021

[44] Brook U, Watemberg N, Geva D. Attitude and knowledge of attention deficit hyperactivity disorder and learning disability among high school teachers. Patient Educ Couns. 2000;40(3):247–52. doi: 10.1016/s0738-3991(99)00080-4 10838003

[45] Rodrigo MDA, Perera D, Eranga VP, Williams SS, Kuruppuarachchi KALA. The knowledge and attitude of primary school teachers in Sri Lanka towards childhood attention deficit hyperactivity disorder. Ceylon Med J. 2011;56(2):51–4. doi: 10.4038/cmj.v56i2.3108 21789864

[46] Flaherty E, Sturm T, Farries E. The conspiracy of Covid-19 and 5G: Spatial analysis fallacies in the age of data democratization. Soc Sci Med. 2022;293:114546. doi: 10.1016/j.socscimed.2021.114546 34954674 PMC8576388

[47] Dudley MZ, Salmon DA, Halsey NA, Orenstein WA, Limaye RJ, O’Leary ST. The Clinician’s Vaccine Safety Resource Guide: Optimizing Prevention of Vaccine-Preventable Diseases Across the Lifespan. 1st ed. Springer International Publishing. 2018.

[48] Kirmayer LJ. Beyond the “new cross-cultural psychiatry”: cultural biology, discursive psychology and the ironies of globalization. Transcult Psychiatry. 2006;43(1):126–44. doi: 10.1177/1363461506061761 16671396

